# Clinical utility of square-wave jerks in neurology and psychiatry

**DOI:** 10.3389/fopht.2023.1302651

**Published:** 2024-01-04

**Authors:** Athena Zachou, Georgios Armenis, Ioannis Stamelos, Eirini Stratigakou-Polychronaki, Fotios Athanasopoulos, Evangelos Anagnostou

**Affiliations:** Department of Neurology, University of Athens, Eginition Hospital, Athens, Greece

**Keywords:** square-wave jerks, eye movements, fixation, saccadic intrusions, progressive supranuclear palsy, cerebellar ataxia

## Abstract

Human eye fixation is steadily interrupted by small, physiological or abnormal, eye movements. Square-wave jerks (SWJ) are the most common saccadic intrusion which can be readily seen at the bedside and also quantified using oculographic techniques. Various neurological, neuropsychiatric and psychiatric disorders display abnormal fixational eye movement patterns characterized by frequent SWJ. For the clinician, SWJ are particularly important because they can be readily observed at the bedside. Here, we will discuss the pathological conditions that present with SWJ and explore the expanding body of literature suggesting that SWJ may serve as a potential indicator for various clinical conditions.

## 1 Introduction

Foveate animals, including humans, can look quietly at a target for prolonged periods of time. This ocular motor condition is called fixation and is based on an active and dynamic eye stabilization system (1). Early on, it became clear that maintained fixation is steadily interrupted by a variety of small, fast and slow, eye displacements (2, 3). Traditionally, these eye movements are divided into two categories: a repertoire of physiological idiosyncratic miniature movements (microsaccades, ocular drift and ocular microtremor) collectively called fixational eye movements (4) and a group of larger, and therefore clinically visible, fixational intrusions comprising SWJ, ocular flutter, opsoclonus, saccadic pulses and macrosaccadic oscillations (5). The distinction of normal fixational eye movements versus abnormal saccadic intrusions, however, might prove to be more of a working hypothesis than an empirical reality. This is particularly true for microsaccades and SWJ, which likely represent different magnitudes across a saccadic continuum extending from fixation to exploration (6). Being easy to assess at the bedside, SWJ are certainly the best-studied saccadic intrusion. Here, we review the measurement methods of SWJ using quantitative oculography and discuss the various neurological and psychiatric conditions that affect the occurrence of SWJ.

## 2 Characteristics, recording and quantification of SWJ

SWJ can be defined as a pair of small (<5 deg) saccades, each of them conjugate. The first one moves the eye away from the fixation point, whereas the second one returns the eye toward the target (corrective saccade), after a short time interval (usually less than 300 ms). SWJ seem to have a strong horizontal preference in humans, something that does not apply to non-human foveate animals, which may show a greater vertical predisposition (7). Macro-SWJ display amplitudes of up to 30 deg and are less commonly observed in clinical practice. Bursts or prolonged series of repetitive, nearly continuous disruptions of fixation are categorized as saccadic oscillations. These may manifest either as SWJ-oscillations or as back-to-back saccadic oscillations without intersaccadic intervals. In the latter case, ocular oscillations are classified as opsoclonus or ocular flutter, depending on whether the oscillations occur in all directions or are restricted to the horizontal plane, respectively.

The genesis of SWJ is still uncertain. A dysfunction of a brainstem oculomotor network is assumed, where the inhibition exerted by the inhibitory burst neurons (IBNs) on the omnipause neurons (OPNs) overcomes the inhibition of the OPNs on the IBNs. Subsequently a short burst of activity appears in the excitatory burst neurons that produces a small saccade. In turn, this produces a small retinal error that is detected in the superior colliculus (SC) which results in a second saccade in the opposite direction, completing a SWJ (8). The cerebellum, basal ganglia, and cortical regions are directly and indirectly interconnected within this network, thereby influencing its functionality (9).

In order to detect and quantify SWJ, precise eye position data has to be obtained. Out of various eye tracking methods, two are the most commonly used for these purposes: Magnetic Field/Scleral Search Coil (SSC) and Video-Oculography (VOG). The former, albeit more invasive and not as well tolerated, is considered more precise and accurate than the latter. However, they have both shown adequate performance in detecting saccades of small magnitude. Temporal resolution is highest for SSC (500+ Hz), but high-end VOG systems can reach frequencies of 400 Hz. Other recording methods, such as Infrared Reflection and DC-electrooculography, are no longer widely used for SWJ detection (10).

Experiments typically consist of subjects viewing a small target on a screen and being asked to maintain fixation for 10-120 seconds. The head is restrained by a forehead-chin headrest. Fixation duration, target shape and size, screen-to-cornea distance and other environmental conditions at the time of the experiment (e.g. lighting) may differ according to the goals of the respective study. In order to detect SWJ from large amounts of data, automated and objective algorithms have been developed. A common approach is to first detect all saccades, using methods reliant on eye movement velocity (11). Saccades are then filtered based on amplitude. Subsequently, pairs of consecutive saccades that are separated by an appropriate intersaccadic interval and display approximately opposite directions and similar magnitudes are classified as SWJ (12). Another approach that has been used is the creation of an SWJ index, incorporating the above metrics in a continuous variable between 0 and 1, and then comparing it to the “ideal SWJ”. Index values above a specified threshold are considered to be SWJ (7).

## 3 SWJ in neurological and psychiatric disorders

### 3.1 SWJ in cerebellar, brainstem and basal ganglia disorders

**Figure 1:**
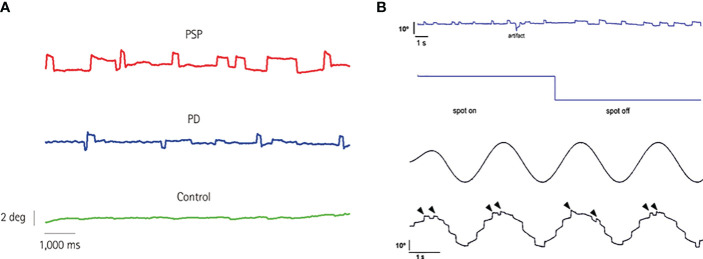
**(A)** Eye position while fixating an LED straight ahead for 10 s in three subjects of the same age (69 years): a PSP patient, a PD patient, and a control subject. Note the increased rate of square-wave jerks in the PSP patient. PD, Parkinson’s disease; PSP, progressive supranuclear palsy. From Anagnostou et al. (9), with permission by the “Korean Neurological Association”. This figure is published in “Anagnostou E, Karavasilis E, Potiri I, Constantinides V, Efstathopoulos E, Kapaki E, Potagas C. A Cortical Substrate for Square-Wave Jerks in Progressive Supranuclear Palsy. J Clin Neurol 2020;16: 37-45”, Copyright Korean Neurological Association. **(B)** 60-year-old man with Langerhans’ cell histiocytosis and cerebellar involvement. Continuous SWJ during straight ahead fixation as well as after removal of visual fixation (upper panel). The average frequency of occurrence was 40.8 min^−1^ and remained unchanged after turning the fixation spot off. In the latter case, however, prolonged off-center fixation periods were demonstrated making square-wave intrusions appear wider. Typical pathological staircase appearance of smooth eye tracking in the same subject due to numerous catch-up saccades (lower panel). Notably, SWJ continue to occur during smooth pursuit (arrowheads). From Anagnostou et al. (13), with permission by “Elsevier”. This figure is published in “Anagnostou E, Papageorgiou SG, Potagas C, Alexakis T, Kalfakis N, Anastasopoulos D. Square-wave jerks and smooth pursuit impairment as subtle early signs of brain involvement in Langerhans’ cell histiocytosis. Clin Neurol Neurosurg 2008;110: 286-290.”, Copyright Elsevier.

Progressive supranuclear palsy (PSP) and cerebellar ataxias are perhaps the most widely recognized neurological disorders in which SWJ play a prominent role in their phenotype (**Figure 1A**). Early on, Troost et al. recognized that SWJ were the second most prevalent ocular motor disorder in PSP after saccadic slowing (14). Subsequently, several clinical and oculographic studies have confirmed and quantified the presence of SWJ in PSP (9, 15–20)]. The occurrence of >10 SWJ/min with amplitudes up to 4 deg, have been recently included in the clinical diagnostic criteria of PSP by the Movement Disorder Society (21). While more prevalent in PSP, SWJ also occur, albeit less consistently, in various other movement disorders (22–28), with Parkinson’s disease being the most characteristic among them (9, 29–31).

Among cerebellar disorders, Friedreich’s ataxia (FA) is the most thoroughly studied with regard to SWJ (32). Ribaï et al. followed 37 FA patients prospectively for 7 years using eye movement recordings. These authors found a high frequency of SWJ in all of them at the beginning of the study, with a further increased rate at the end of it (33). In 2008, Fahey et al. investigated SWJ in 15 Friedreich’s ataxia (FA) patients under target-on and target-off conditions. They observed longer and larger SWJ in the target-off condition. Younger patients exhibited more fixation instability with more frequent and shorter SWJ in both conditions; no differences in the total number of SWJ between ambulant and non-ambulant participants were found, but the latter group exhibited significantly more macro-SWJ. Interestingly, in almost half of the patients, oblique SWJ with a prominent vertical component, as well as occasional pure vertical SWJ, were recorded (34).

SWJ have been described in many spinocerebellar ataxias (SCA), including SCA1, SCA2, SCA8, SCA10, SCA14, SCA20, SCA21, SCA25, SCA27, SCA29, SCA37, SCA46, and DRLPA, although they are more noticeable in SCA3 and SCA6 (35, 36). Bürk et al. detected SWJ in 30% of SCA3 patients (37), whereas another study reported a percentage of approximately 55% for SCA3 patients; SCA1, SCA2, and SCA6 percentages were found lower, around 20% (38). Similarly, Moscovich et al. found SWJ in 23% of SCA3 patients and 17% of SCA6 patients (39). In 2017, Wu et al. investigated ocular movements in SCA3 and found a high frequency of SWJ in both symptomatic patients (n=44) and SCA3 carriers (n=12) (40). Additionally, Bour et al. recorded downbeat nystagmus accompanied by horizontal SWJ (bow-tie nystagmus) in the majority of SCA6 patients they recruited (n=4/6). Similar oculomotor findings (bow-tie nystagmus) were also observed in patients with familial cortical myoclonic tremor with epilepsy (FCMTE) (n=4/6) (41).

SWJ along with gaze-evoked and/or spontaneous nystagmus are common features of Ataxia-Telangiectasia (42). High SWJ frequency and amplitude have been also found in patients with Ataxia with Oculomotor apraxia type 2 (43). Pronounced oculomotor abnormalities with frequent SWJ have been observed in patients with chorea-acanthocytosis, who have heterozygous VPS13A mutations and basal ganglia degeneration, when compared to normal controls (25). Likewise, SWJ have been reported in adult-onset Alexander disease (44) and X-linked ataxia (45).

Paraneoplastic cerebellar degeneration is associated with a frequent occurrence of SWJ as described in cases of anti-Hu and anti-CV2/CRMP5 cerebellar ataxia associated with Small-Cell Lung Cancer (SCLC) (46, 47). Additionally, SWJ are observed in paraneoplastic Stiff-person syndrome with anti-amphiphysin antibodies, which can be linked to malignancies like SCLC, breast cancer, and Hodgkin’s Lymphoma (47). Similarly, frequent SWJ can be found in cerebellar ataxia due to autoimmune mechanisms, whether or not autoantibodies are detected in serum or cerebrospinal fluid (CSF). This includes conditions like anti-GAD Stiff-person syndrome (47) or anti-Sj/ITPR1 and anti-NMDA cerebellar ataxia (48). High frequency SWJ (“square wave oscillations”) have been reported in a case of anti-GAD cerebellar ataxia accompanied by autoimmune thyroiditis (49). They have also been described in a patient with steroid-responsive encephalopathy with autoimmune thyroiditis presenting with pure cerebellar ataxia (50) and in a case of a SCLC patient with pembrolizumab therapy-induced encephalitis (51).

A high occurrence of SWJs was found in a case of Langerhans’ cell histiocytosis (LCH) with central nervous system involvement (13) (**Figure 1B**) and has also been described in Creutzfeldt-Jacob disease, presenting with progressive cerebellar ataxia, oculomotor abnormalities, mental impairment and hyper-intensities in the basal ganglia and thalami (52). In Arnold-Chiari malformation type 1, SWJ along with downbeat nystagmus have been described, but only after strabismus surgery (53). In contrast, Chiari type 2 malformation is not associated with an increased SWJ rate (54).

Finally, oculomotor recordings in patients with essential tremor have revealed an increased rate of SWJs, along with increased saccade latency and decreased saccade peak velocity when compared with normal controls. These findings appear to be unrelated to the duration, severity, or treatment of the disease (55).

### 3.2 SWJ in dementias

It has long been reported that SWJ occur in patients with acute or chronic focal cerebral lesions, regardless of the lesion’s location. The amplitude of these SWJ is typically lower than that observed in cerebellar diseases (56).

Typically, SWJ are generated during visual fixation (VF) and suppressed in darkness. However, in Alzheimer’s disease (AD), SWJ occur more frequently in low-light conditions than during VF. These SWJ without VF are associated with increased cortical dysfunction (57).

Nonetheless, it has been reported that patients with AD and control subjects do not differ in the rate of intrusive saccades during VF at baseline. Only AD patients exhibit a progressive increase over an 18-month follow-up period, and this increase correlates with heightened dementia severity. However, it is not specified whether this phenomenon holds true for SWJ, which are paired intrusive saccades characterized by equal amplitude and opposite direction (58).

In another publication, healthy controls and young onset Alzheimer’s disease patients (YOAD) also showed no significant difference in the average number of SWJ. However, YOAD patients exhibited a higher frequency of large intrusive saccades and had shorter periods of fixation compared to healthy controls (59).

A slightly different scenario is presented in a study by Shakespeare et al. (60). Patients with typical AD demonstrated an elevated rate of SWJ during attempted VF in comparison to healthy controls. Furthermore, the SWJ rate was found to be associated with a decrease in cerebellar grey matter volume. On the other hand, patients with posterior cortical atrophy, a variant of AD, exhibited increased occurrence of large unpaired saccadic intrusions, which correlated with reduction in cortical thickness.

Small SWJ are defined as paired microsaccades with equal amplitude and opposite directions. Microsaccades in individuals with AD or mild cognitive impairment tend to exhibit a more oblique trajectory when compared to those in healthy controls (61). A high frequency of small SWJ has also been observed in the behavioral variant frontotemporal dementia group compared to age-matched controls. Neural correlates were identified in the orbitofrontal and ventromedial prefrontal cortices, as well as the striatum.

### 3.3 SWJ in psychiatry

Eye movement studies in psychiatric diseases are more sparse then those in neurologic disorders. Impairments in different oculomotor paradigms have been reported in major depressive disorder (MDD) (62, 63), in bipolar disorder (BD) (62, 64), in obsessive compulsive disorder (OCD) (65), in anorexia nervosa (AN) (66) and especially in schizophrenia (SCZ) (67–69) with some researchers considering them as potential biomarkers (63, 70–72) while others remain more skeptical (68, 73).

SWJ in psychiatric diseases, however, have been less frequently studied, and this not always in dedicated visual fixation paradigms. Regarding SCZ most of the studies have found no differences between SCZ patients and health controls (HC) (67, 74–78). However, one study (79) reported increased frequency in SWJ during smooth pursuit eye movements, while others have even reported lower SWJ rates in patients (80, 81). Clearly, differences in methodological approaches account for these discrepancies. More recently, Levy et al. reported that SWJ frequency was one of the variables that discriminated between subjects with normal and subjects with abnormal smooth pursuit performance (in a mixed group of HC and schizophrenic patients). Hence, the presence of another ocular motor dysfunction (i.e. smooth pursuit) rather than SCZ itself appears critical with respect to the occurrence of SWJ (82).

Data on SWJ in affective disorders are not less contradictory. Sweeney et al. found that patients with BD presented higher rates of SWJ compared to SCZ patients but had no differences compared to controls (81). Flechtner et al. compared the number of SWJ produced by SCZ patients, patients with affective disorders (both MDD and BP) and controls in a smooth pursuit paradigm and reported a trend for patients with affective disorders to perform more SWJ (77). On the other hand, Friedman et al. did not spot any differences between SCZ patients, patients with affective disorders and controls (76, 80). Finally, Sweeney et al. reported increased SWJ rates in MDD patients using a visual fixation task in contrast to the previous studies (83).

Concerning patients with OCD, Sweeney et al. reported increased frequency of SWJ during a smooth pursuit task (84). On the contrary, other studies (85–87) found no increase in the frequency of SWJ in OCD patients compared to controls. Moreover, Pallanti et al. observed SWJ in three anorectic patients while they were absent in controls (88). In a study by Phillipou et al. it was shown that AN patients made SWJ at a higher rate compared to controls which was also negatively correlated with anxiety. Also, 87.7% of AN subjects and 95.5% of healthy participants were properly classified based on SWJ and anxiety scores (89).

## 4 Discussion

Square Wave Jerks (SWJ) are an especially valuable ocular motor sign due to their ease of detection during bedside physical examinations. However, they often go unnoticed and receive far less attention compared to larger eye movements like saccades and smooth pursuit among general neurologists.

Dedicated studies on SWJ are still relatively scarce, but accumulating evidence underscores their significant relevance in the ocular motor characteristics of movement disorders and cerebellar ataxias. Certainly, PSP and cerebellar syndromes of various etiologies (degenerative, autoimmune, paraneoplastic) stand out as the most extensively studied and well-established central nervous system disorders associated with saccadic intrusions. When it comes to neurodegenerative dementias, there exist somewhat conflicting results. Most studies focus on patients with AD, with some demonstrating an increase in SWJ rates compared to age-matched controls, while others show normal rates. Clearly, methodological variations and, more importantly, diagnostic uncertainties may account for these discrepancies. The inclusion of more homogeneous diagnostic groups based on cerebrospinal fluid biomarkers rather than relying solely on the neuropsychological profile might enable more robust conclusions regarding the role of SWJ in dementia. The same applies to psychiatric disorders, where the concept of drug naivety may be of particular importance in designing methodologically robust studies.

Many clinicians consider SWJ to be rather nonspecific, as they can occur in otherwise healthy elderly individuals, particularly when the rest of the oculomotor examination is normal. The number of SWJ per minute might therefore be a critical parameter given the fact that the occurrence of SWJs is not a binary on-off phenomenon distinguishing health from disease but rather a continuum. To establish a valid clinical criterion, thresholding, particularly in terms of SWJ frequency, is essential. **Table 1** offers an overview of SWJ rates as reported in the available literature. Only studies that reported mean SWJ frequencies, which can be converted into rates per minute, were included, while single case reports were excluded.

**Table 1:** Summary table of SWJ rates in neurological and psychiatric disorders.

Disorder	Study	Diagnosis	No. of Patients	Mean SWJ rate (SWJ/min)
Brainstem/Cerebellar/Basal Ganglia	Rascol et al. (15),	PSP	7	54
	Otero-Millan et al. (8),	PSP	10	48
	Anagnostou et al. (9),	PSP	20	33.5 (fixation on) 22.5 (fixation off)
	Pagonabarraga et al. (19),	PSP	65	22.8
	Becker et al., (20)	PSP	50	31.2
	White et al. (29),	PD	14	52
	Rascol et al. (15),	PD	13	45
	Anagnostou et al. (9),	PD	12	10.3 (fixation on) 13.3 (fixation off)
	Pagonabarraga et al. (19),	PD	25	1.2
	Bonnet et al. (27),	Ephedrone-induced Parkinsonism	28	6.8
	Ribaï et al. (33),	Friedreich ataxia	37	36 (first assessment) 54 (7 years later)
	Salman et al. (54),	Chiari malformation type 2	21	3.5
	Gitchel et al. (55),	Essential Tremor	60	26.9
	Wu et al. (40),	SCA3	44 patients 12 pre-clinical carriers	47 32
Dementia	Nakamagoe et al. (57),	Alzheimer’s Disease	15	10.4 (fixation on) 20 (fixation off)
	Pavisic et al. (59),	Alzheimer’s Disease (young onset), Posterior Cortical Atrophy (young onset)	26, 10	6.2, 4.9
	Shakespeare et al. (60),	Alzheimer’s Disease, Posterior Cortical Atrophy	17, 20	36, 18.9
	Kapoula et al. (61),	Alzheimer’s Disease, Mild Cognitive Impairment	18, 15	2.0, 2.2
	Russell et al., 2021 (90),	Frontotemporal degeneration (behavioral variant)	19	18.4
Psychiatric	Nickoloff et al. (85),	Obsessive-compulsive disorder	8	3.4
	Sweeney et al. (84),	Obsessive-compulsive disorder	17	7.5
	Campion et al. (75),	Schizophrenia	46 (13 drug-naïve, 20 chronic, 13 residual)	12
	Friedman et al. (80),	Schizophrenia, Affectives	23, 16	7.2, 5.0
	Sweeney et al. (81),	Schizophrenia, Bipolar disorder, Major depressive disorder	101, 17, 13	2.8, 7.5, 3.5
	Friedman et al. (76),	Schizophrenia, Affectives	26, 14	19.8, 24.6
	Flechtner et al. (77),	Schizophrenia, Affectives	43, 34	9.6, 14.7
	Levy et al., (82)	Schizophrenia	43	6
	Phillipou et al. (89),	Anorexia nervosa	22	11.8

There are still important questions awaiting answers concerning the neuronal mechanisms that govern fixation in humans. To gain a deeper understanding of the mechanisms underlying saccadic intrusions in neurological and psychiatric disorders, it would be beneficial to perform a more detailed oculographic categorization of SWJ waveforms, considering factors such as amplitude, direction, and intersaccadic intervals. Combining clinical assessments with quantitative oculographic analysis and both structural and functional neuroimaging approaches is expected to provide further insights into the pathophysiology and clinical significance of ocular oscillations in various disease categories.

## Author contributions

AZ: Writing – original draft, Writing – review & editing. GA: Writing – original draft, Writing – review & editing. IS: Writing – original draft, Writing – review & editing. ES: Writing – original draft, Writing – review & editing. FA: Writing – original draft, Writing – review & editing. EA: Writing – original draft, Writing – review & editing.

## References

[1] Robinson DA. Eye stabilization. Prog Brain Res (2022) 267(1):379–90. doi: 10.1016/bs.pbr.2021.10.018 35074063

[2] Ditchburn RW Ginsborg BL. Involuntary eye movements during fixation. J Physiol (1953) 119(1):1–17. doi: 10.1113/jphysiol.1953.sp004824 13035713 PMC1393034

[3] Steinman RM Haddad GM Skavenski AA Wyman D. Miniature eye movement. Science (1973) 181(4102):810–9. doi: 10.1126/science.181.4102.810 4198903

[4] Martinez-Conde S Macknik SL Hubel DH. The role of fixational eye movements in visual perception. Nat Rev Neurosci (2004) 5(3):229–40. doi: 10.1038/nrn1348 14976522

[5] Lemos J Eggenberger E. Saccadic intrusions: review and update. Curr Opin Neurol (2013) 26(1):59–66. doi: 10.1097/WCO.0b013e32835c5e1d 23302805

[6] Otero-Millan J Macknik SL Langston RE Martinez-Conde S. An oculomotor continuum from exploration to fixation. Proc Natl Acad Sci U S A (2013) 110(15):6175–80. doi: 10.1073/pnas.1222715110 PMC3625326 23533278

[7] Costela FM Otero-Millan J McCamy MB Macknik SL Di Stasi LL Rieiro H. Characteristics of spontaneous square-wave jerks in the healthy macaque monkey during visual fixation. PLoS One (2015) 10(6):e0126485. doi: 10.1371/journal.pone.0126485 26067994 PMC4466238

[8] Otero-Millan J Macknik SL Serra A Leigh RJ Martinez-Conde S. Triggering mechanisms in microsaccade and saccade generation: a novel proposal. Ann N Y Acad Sci (2011) 1233:107–16. doi: 10.1111/j.1749-6632.2011.06177.x 21950983

[9] Anagnostou E Karavasilis E Potiri I Constantinides V Efstathopoulos E Kapaki E. A cortical substrate for square-wave jerks in progressive supranuclear palsy. J Clin Neurol (2020) 16(1):37–45. doi: 10.3988/jcn.2020.16.1.37 31942756 PMC6974821

[10] Eggert T. Eye movement recordings: methods. Dev Ophthalmol (2007) 40:15–34. doi: 10.1159/000100347 17314477

[11] Engbert R Kliegl R. Microsaccades uncover the orientation of covert attention. Vision Res (2003) 43(9):1035–45. doi: 10.1016/S0042-6989(03)00084-1 12676246

[12] Salman MS Sharpe JA Lillakas L Steinbach MJ. Square wave jerks in children and adolescents. Pediatr Neurol (2008) 38(1):16–9. doi: 10.1016/j.pediatrneurol.2007.08.011 18054687

[13] Anagnostou E Papageorgiou SG Potagas C Alexakis T Kalfakis N Anastasopoulos D. Square-wave jerks and smooth pursuit impairment as subtle early signs of brain involvement in Langerhans' cell histiocytosis. Clin Neurol Neurosurg (2008) 110(3):286–90. doi: 10.1016/j.clineuro.2007.10.008 18078708

[14] Troost BT Daroff RB. The ocular motor defects in progressive supranuclear palsy. Ann Neurol (1977) 2(5):397–403. doi: 10.1002/ana.410020509 617579

[15] Rascol O Sabatini U Simonetta-Moreau M Montastruc JL Rascol A Clanet M. Square wave jerks in parkinsonian syndromes. J Neurol Neurosurg Psychiatry (1991) 54(7):599–602. doi: 10.1136/jnnp.54.7.599 1895124 PMC1014429

[16] Rivaud-Péchoux S Vidailhet M Gallouedec G Litvan I Gaymard B Pierrot-Deseilligny C. Longitudinal ocular motor study in corticobasal degeneration and progressive supranuclear palsy. Neurology (2000) 54(5):1029–32. doi: 10.1212/WNL.54.5.1029 10720270

[17] Otero-Millan J Serra A Leigh RJ Troncoso XG Macknik SL Martinez-Conde S. Distinctive features of saccadic intrusions and microsaccades in progressive supranuclear palsy. J Neurosci (2011) 31(12):4379–87. doi: 10.1523/JNEUROSCI.2600-10.2011 PMC3111217 21430139

[18] Otero-Millan J Schneider R Leigh RJ Macknik SL Martinez-Conde S. Saccades during attempted fixation in parkinsonian disorders and recessive ataxia: from microsaccades to square-wave jerks. PLoS One (2013) 8(3):e58535. doi: 10.1371/journal.pone.0058535 23516502 PMC3596296

[19] Pagonabarraga J Horta-Barba A Busteed L Bejr-Kasem H Illán-Gala I Aracil-Bolaños I. Quantitative evaluation of oculomotor disturbances in progressive supranuclear palsy. Parkinsonism Relat Disord (2021) 85:63–8. doi: 10.1016/j.parkreldis.2021.03.002 33744691

[20] Becker W Behler A Vintonyak O Kassubek J. Patterns of small involuntary fixation saccades (SIFSs) in different neurodegenerative diseases: the role of noise. Exp Brain Res (2023) 241(7):1821–33. doi: 10.1007/s00221-023-06633-6 PMC10348992 37247026

[21] Höglinger GU Respondek G Stamelou M Kurz C Josephs KA Lang AE. Clinical diagnosis of progressive supranuclear palsy: The movement disorder society criteria. Mov Disord (2017) 32(6):853–64. doi: 10.1002/mds.26987 PMC5516529 28467028

[22] Bollen E Reulen JP Den Heyer JC van der Kamp W Roos RA Buruma OJ. Horizontal and vertical saccadic eye movement abnormalities in Huntington's chorea. J Neurol Sci (1986) 74(1):11–22. doi: 10.1016/0022-510X(86)90187-5 2941523

[23] Aramideh M Bour LJ Koelman JH Speelman JD Ongerboer de Visser BW. Abnormal eye movements in blepharospasm and involuntary levator palpebrae inhibition. Clinical and pathophysiological considerations. Brain (1994) 117(Pt 6):1457–74. doi: 10.1093/brain/117.6.1457 7820580

[24] Egan RA Weleber RG Hogarth P Gregory A Coryell J Westaway SK. Neuro-ophthalmologic and electroretinographic findings in pantothenate kinase-associated neurodegeneration (formerly Hallervorden-Spatz syndrome). Am J Ophthalmol (2005) 140(2):267–74. doi: 10.1016/j.ajo.2005.03.024 PMC2169522 16023068

[25] Gradstein L Danek A Grafman J Fitzgibbon EJ. Eye movements in chorea-acanthocytosis. Invest Ophthalmol Vis Sci (2005) 46(6):1979–87. doi: 10.1167/iovs.04-0539 15914612

[26] Anderson T Luxon L Quinn N Daniel S David Marsden C Bronstein A. Oculomotor function in multiple system atrophy: clinical and laboratory features in 30 patients. Mov Disord (2008) 23(7):977–84. doi: 10.1002/mds.21999 18383533

[27] Bonnet C Rusz J Megrelishvili M Sieger T Matoušková O Okujava M. Eye movements in ephedrone-induced parkinsonism. PLoS One (2014) 9(8):e104784. doi: 10.1371/journal.pone.0104784 25117825 PMC4130591

[28] Khan AO AlDrees A Elmalik SA Hassan HH Michel M Stevanin G. Ophthalmic features of PLA2G6-related paediatric neurodegeneration with brain iron accumulation. Br J Ophthalmol (2014) 98(7):889–93. doi: 10.1136/bjophthalmol-2013-304527 24522175

[29] White OB Saint-Cyr JA Tomlinson RD Sharpe JA. Ocular motor deficits in Parkinson's disease. II. Control of the saccadic and smooth pursuit systems. Brain (1983) 106(Pt 3):571–87. doi: 10.1093/brain/106.3.571 6640270

[30] Pinnock RA McGivern RC Forbes R Gibson JM. An exploration of ocular fixation in Parkinson's disease, multiple system atrophy and progressive supranuclear palsy. J Neurol (2010) 257(4):533–9. doi: 10.1007/s00415-009-5356-3 19847469

[31] Shaikh AG Xu-Wilson M Grill S Zee DS. 'Staircase' square-wave jerks in early Parkinson's disease. Br J Ophthalmol (2011) 95(5):705–9. doi: 10.1136/bjo.2010.179630 20693560

[32] Baloh RW Konrad HR Honrubia V. Vestibulo-ocular function in patients with cerebellar atrophy. Neurology (1975) 25(2):160–8. doi: 10.1212/WNL.25.2.160 1078721

[33] Ribaï P Pousset F Tanguy ML Rivaud-Pechoux S Le Ber I Gasparini F. Neurological, cardiological, and oculomotor progression in 104 patients with Friedreich ataxia during long-term follow-up. Arch Neurol (2007) 64(4):558–64. doi: 10.1001/archneur.64.4.558 17420319

[34] Fahey MC Cremer PD Aw ST Millist L Todd MJ White OB. Vestibular, saccadic and fixation abnormalities in genetically confirmed Friedreich ataxia. Brain (2008) 131(Pt 4):1035–45. doi: 10.1093/brain/awm323 18238798

[35] Rosini F Pretegiani E Battisti C Dotti MT Federico A Rufa A. Eye movement changes in autosomal dominant spinocerebellar ataxias. Neurol Sci (2020) 41(7):1719–34. doi: 10.1007/s10072-020-04318-4 32130555

[36] Zachou A Palaiologou D Kanavakis E Anagnostou E. Retrocollis as the cardinal feature in a *de novo* ITRP1 variant. Brain Dev (2022) 44(5):347–52. doi: 10.1016/j.braindev.2022.01.005 35148930

[37] Bürk K Fetter M Abele M Laccone F Brice A Dichgans J. Autosomal dominant cerebellar ataxia type I: oculomotor abnormalities in families with SCA1, SCA2, and SCA3. J Neurol (1999) 246(9):789–97. doi: 10.1007/s004150050456 10525976

[38] Kim JS Kim JS Youn J Seo DW Jeong Y Kang JH. Ocular motor characteristics of different subtypes of spinocerebellar ataxia: distinguishing features. Mov Disord (2013) 28(9):1271–7. doi: 10.1002/mds.25464 23609488

[39] Moscovich M Okun MS Favilla C Figueroa KP Pulst SM Perlman S. Clinical evaluation of eye movements in spinocerebellar ataxias: a prospective multicenter study. J Neuroophthalmol (2015) 35(1):16–21. doi: 10.1097/WNO.0000000000000167 25259863 PMC4675453

[40] Wu C Chen DB Feng L Zhou XX Zhang JW You HJ. Oculomotor deficits in spinocerebellar ataxia type 3: Potential biomarkers of preclinical detection and disease progression. CNS Neurosci Ther (2017) 23(4):321–8. doi: 10.1111/cns.12676 PMC6492748 28195427

[41] Bour LJ van Rootselaar AF Koelman JH Tijssen MA. Oculomotor abnormalities in myoclonic tremor: a comparison with spinocerebellar ataxia type 6. Brain (2008) 131(Pt 9):2295–303. doi: 10.1093/brain/awn177 18687731

[42] Shaikh AG Marti S Tarnutzer AA Palla A Crawford TO Straumann D. Gaze fixation deficits and their implication in ataxia-telangiectasia. J Neurol Neurosurg Psychiatry (2009) 80(8):858–64. doi: 10.1136/jnnp.2008.170522 19357126

[43] Clausi S De Luca M Chiricozzi FR Tedesco AM Casali C Molinari M. Oculomotor deficits affect neuropsychological performance in oculomotor apraxia type 2. Cortex (2013) 49(3):691–701. doi: 10.1016/j.cortex.2012.02.007 22480402

[44] Martidis A Yee RD Azzarelli B Biller J. Neuro-ophthalmic, radiographic, and pathologic manifestations of adult-onset Alexander disease. Arch Ophthalmol (1999) 117(2):265–7. doi: 10.1001/archopht.117.2.265 10037578

[45] Verhagen WI Huygen PL Arts WF. Multi-system signs and symptoms in X-linked ataxia carriers. J Neurol Sci (1996) 140(1-2):85–90. doi: 10.1016/0022-510X(96)00116-5 8866431

[46] Schiff ND Moore DF Winterkorn JM. Predominant downgaze ophthalmoparesis in anti-Hu encephalomyelitis. J Neuroophthalmol (1996) 16(4):302–3. 8956170

[47] Ko MW Dalmau J Galetta SL. Neuro-ophthalmologic manifestations of paraneoplastic syndromes. J Neuroophthalmol (2008) 28(1):58–68. doi: 10.1097/WNO.0b013e3181677fcc 18347462

[48] Chapman WJ Broderick J Ferioli S. Co-occurrence of Sj/ITPR1 and NMDA antibodies: A case report (P2-1.002). Neurology (2022) 98(18 Supplement):1489. doi: 10.1212/WNL.98.18_supplement.1489

[49] Brokalaki C Kararizou E Dimitrakopoulos A Evdokimidis I Anagnostou E. Square-wave ocular oscillation and ataxia in an anti-GAD-positive individual with hypothyroidism. J Neuroophthalmol (2015) 35(4):390–5. doi: 10.1097/WNO.0000000000000275 26035807

[50] Termsarasab P Pitakpatapee Y Frucht SJ Srivanitchapoom P. Steroid-responsive encephalopathy associated with autoimmune thyroiditis (SREAT) presenting with pure cerebellar ataxia. Tremor Other Hyperkinet Mov (N Y) (2018) 8:585. doi: 10.5334/tohm.420 30191089 PMC6125737

[51] Vitt JR Kreple C Mahmood N Dickerson E Lopez GY Richie MB. Autoimmune pancerebellitis associated with pembrolizumab therapy. Neurology (2018) 91(2):91–3. doi: 10.1212/WNL.0000000000005781 PMC6053111 29875219

[52] Chwalisz BK Buchbinder BR Schmahmann JD Samore WR. Case 32-2019: A 70-year-old woman with rapidly progressive ataxia. N Engl J Med (2019) 381(16):1–7. doi: 10.1056/NEJMcpc1909624 31618544

[53] Passo M Shults WT Talbot T Palmer EA. Acquired esotropia. A manifestation of Chiari I malformation. J Clin Neuroophthalmol (1984) 4(3):151–4. 10.3109/01658108409034894 6238049

[54] Salman MS Sharpe JA Lillakas L Dennis M Steinbach MJ. Visual fixation in Chiari type II malformation. J Child Neurol (2009) 24(2):161–5. doi: 10.1177/0883073808322326 PMC3050044 19182152

[55] Gitchel GT Wetzel PA Baron MS. Slowed saccades and increased square wave jerks in essential tremor. Tremor Other Hyperkinet Mov (N Y) (2013) 3. doi: 10.5334/tohm.127 PMC3779821 24116343

[56] Sharpe JA Herishanu YO White OB. Cerebral square wave jerks. Neurology (1982) 32(1):57–62. doi: 10.1212/WNL.32.1.57 7198734

[57] Nakamagoe K Yamada S Kawakami R Koganezawa T Tamaoka A. Abnormal saccadic intrusions with Alzheimer's disease in darkness. Curr Alzheimer Res (2019) 16(4):293–301. doi: 10.2174/1567205016666190311102130 30854969

[58] Bylsma FW Rasmusson DX Rebok GW Keyl PM Tune L Brandt J. Changes in visual fixation and saccadic eye movements in Alzheimer's disease. Int J Psychophysiol (1995) 19(1):33–40. doi: 10.1016/0167-8760(94)00060-R 7790287

[59] Pavisic IM Firth NC Parsons S Rego DM Shakespeare TJ Yong KXX. Eyetracking metrics in young onset Alzheimer's disease: A window into cognitive visual functions. Front Neurol (2017) 8:377. doi: 10.3389/fneur.2017.00377 28824534 PMC5545969

[60] Shakespeare TJ Kaski D Yong KX Paterson RW Slattery CF Ryan NS. Abnormalities of fixation, saccade and pursuit in posterior cortical atrophy. Brain (2015) 138(Pt 7):1976–91. doi: 10.1093/brain/awv103 PMC4572483 25895507

[61] Kapoula Z Yang Q Otero-Millan J Xiao S Macknik SL Lang A. Distinctive features of microsaccades in Alzheimer's disease and in mild cognitive impairment. Age (Dordr) (2014) 36(2):535–43. doi: 10.1007/s11357-013-9582-3 PMC4039256 24037325

[62] Carvalho N Laurent E Noiret N Chopard G Haffen E Bennabi D. Eye movement in unipolar and bipolar depression: A systematic review of the literature. Front Psychol (2015) 6:1809. doi: 10.3389/fpsyg.2015.01809 26696915 PMC4678228

[63] Takahashi J Hirano Y Miura K Morita K Fujimoto M Yamamori H. Eye movement abnormalities in major depressive disorder. Front Psychiatry (2021) 12:673443. doi: 10.3389/fpsyt.2021.673443 34447321 PMC8382962

[64] Wang Y Lyu HL Tian XH Lang B Wang XY St Clair D. The similar eye movement dysfunction between major depressive disorder, bipolar depression and bipolar mania. World J Biol Psychiatry (2022) 23(9):689–702. doi: 10.1080/15622975.2022.2025616 35112653

[65] Jaafari N Rigalleau F Rachid F Delamillieure P Millet B Olié JP. A critical review of the contribution of eye movement recordings to the neuropsychology of obsessive compulsive disorder. Acta Psychiatr Scand (2011) 124(2):87–101. doi: 10.1111/j.1600-0447.2011.01721.x 21631433

[66] Phillipou A Abel LA Gurvich C Castle DJ Rossell SL. Eye movements in anorexia nervosa: State or trait markers? Int J Eat Disord (2020) 53(10):1678–84. doi: 10.1002/eat.23345 32720354

[67] Levy DL Holzman PS Matthysse S Mendell NR. Eye tracking dysfunction and schizophrenia: a critical perspective. Schizophr Bull (1993) 19(3):461–536. doi: 10.1093/schbul/19.3.461 8235455

[68] Smyrnis N Amado I Krebs M-O Sweeney JA. Eye movements in psychiatry. In: Klein C Ettinger U, editors. Eye Movement Research. Springer Nature Cham, Switzerland (2019). p. 703–48.

[69] Wolf A Ueda K Hirano Y. Recent updates of eye movement abnormalities in patients with schizophrenia: A scoping review. Psychiatry Clin Neurosci (2021) 75(3):82–100. doi: 10.1111/pcn.13188 33314465 PMC7986125

[70] Benson PJ Beedie SA Shephard E Giegling I Rujescu D St Clair D. Simple viewing tests can detect eye movement abnormalities that distinguish schizophrenia cases from controls with exceptional accuracy. Biol Psychiatry (2012) 72(9):716–24. doi: 10.1016/j.biopsych.2012.04.019 22621999

[71] Brakemeier S Sprenger A Meyhöfer I McDowell JE Rubin LH Hill SK. Smooth pursuit eye movement deficits as a biomarker for psychotic features in bipolar disorder-Findings from the PARDIP study. Bipolar Disord (2020) 22(6):602–11. doi: 10.1111/bdi.12865 31721386

[72] Lyu H St Clair D Wu R Benson PJ Guo W Wang G. Eye movement abnormalities can distinguish first-episode schizophrenia, chronic schizophrenia, and prodromal patients from healthy controls. Schizophr Bull Open (2023) 4(1):1–11. doi: 10.1093/schizbullopen/sgac076 PMC11207660 39145342

[73] Athanasopoulos F Saprikis OV Margeli M Klein C Smyrnis N. Towards clinically relevant oculomotor biomarkers in early schizophrenia. Front Behav Neurosci (2021) 15:688683. doi: 10.3389/fnbeh.2021.688683 34177483 PMC8222521

[74] Levin S Luebke A Zee DS Hain TC Robinson DA Holzman PS. Smooth pursuit eye movements in schizophrenics: quantitative measurements with the search-coil technique. J Psychiatr Res (1988) 22(3):195–206. doi: 10.1016/0022-3956(88)90005-2 3225789

[75] Campion D Thibaut F Denise P Courtin P Pottier M Levillain D. SPEM impairment in drug-naive schizophrenic patients: evidence for a trait marker. Biol Psychiatry (1992) 32(10):891–902. doi: 10.1016/0006-3223(92)90178-3 1361365

[76] Friedman L Jesberger JA Siever LJ Thompson P Mohs R Meltzer HY. Smooth pursuit performance in patients with affective disorders or schizophrenia and normal controls: analysis with specific oculomotor measures, RMS error and qualitative ratings. Psychol Med (1995) 25(2):387–403. doi: 10.1017/S003329170003628X 7675926

[77] Flechtner KM Steinacher B Sauer R Mackert A. Smooth pursuit eye movements in schizophrenia and affective disorder. Psychol Med (1997) 27(6):1411–9. doi: 10.1017/S0033291797005709 9403912

[78] Boudet C Denise P Bocca ML Chabot B Abadie P Brazo P. Eye tracking disorders in schizophrenic patients and their parents. Encephale (2001) 27(6):551–8. 11865562

[79] Levin S Jones A Stark L Merrin EL Holzman PS. Identification of abnormal patterns in eye movements of schizophrenic patients. Arch Gen Psychiatry (1982) 39(10):1125–30. doi: 10.1001/archpsyc.1982.04290100005002 6127062

[80] Friedman L Abel LA Jesberger JA Malki A Meltzer HY. Saccadic intrusions into smooth pursuit in patients with schizophrenia or affective disorder and normal controls. Biol Psychiatry (1992) 31(11):1110–8. doi: 10.1016/0006-3223(92)90155-S 1525275

[81] Sweeney JA Clementz BA Haas GL Escobar MD Drake K Frances AJ. Eye tracking dysfunction in schizophrenia: characterization of component eye movement abnormalities, diagnostic specificity, and the role of attention. J Abnorm Psychol (1994) 103(2):222–30. doi: 10.1037/0021-843X.103.2.222 8040491

[82] Levy DL Lajonchere CM Dorogusker B Min D Lee S Tartaglini A. Quantitative characterization of eye tracking dysfunction in schizophrenia. Schizophr Res (2000) 42(3):171–85. doi: 10.1016/S0920-9964(99)00122-X 10785576

[83] Sweeney JA Strojwas MH Mann JJ Thase ME. Prefrontal and cerebellar abnormalities in major depression: evidence from oculomotor studies. Biol Psychiatry (1998) 43(8):584–94. doi: 10.1016/S0006-3223(97)00485-X 9564443

[84] Sweeney JA Palumbo DR Halper JP Shear MK. Pursuit eye movement dysfunction in obsessive-compulsive disorder. Psychiatry Res (1992) 42(1):1–11. doi: 10.1016/0165-1781(92)90034-Z 1603877

[85] Nickoloff SE Radant AD Reichler R Hommer DW. Smooth pursuit and saccadic eye movements and neurological soft signs in obsessive-compulsive disorder. Psychiatry Res (1991) 38(2):173–85. doi: 10.1016/0165-1781(91)90042-N 1754630

[86] Tien AY Pearlson GD Machlin SR Bylsma FW Hoehn-Saric R. Oculomotor performance in obsessive-compulsive disorder. Am J Psychiatry (1992) 149(5):641–6. doi: 10.1176/ajp.149.5.641 1575255

[87] Pallanti S Grecu LM Gangemi PF Massi S Parigi A Arnetoli G. Smooth-pursuit eye movement and saccadic intrusions in obsessive-compulsive disorder. Biol Psychiatry (1996) 40(11):1164–72. doi: 10.1016/S0006-3223(95)00607-9 8931920

[88] Pallanti S Quercioli L Zaccara G Ramacciotti AB Arnetoli G. Eye movement abnormalities in anorexia nervosa. Psychiatry Res (1998) 78(1-2):59–70. doi: 10.1016/S0165-1781(97)00139-X 9579703

[89] Phillipou A Rossell SL Castle DJ Gurvich C Abel LA. Square wave jerks and anxiety as distinctive biomarkers for anorexia nervosa. Invest Ophthalmol Vis Sci (2014) 55(12):8366–70. doi: 10.1167/iovs.14-15807 25468894

[90] Russell LL Greaves CV Convery RS Bocchetta M Warren JD Kaski D. Eye movements in frontotemporal dementia: Abnormalities of fixation, saccades and anti-saccades. Alzheimers Dement (N Y) (2021) 7(1):e12218. doi: 10.3389/fpsyg.2015.01809 35005203 PMC8719345

